# Spatio-temporal patterns of scrub typhus in mainland China, 2006-2017

**DOI:** 10.1371/journal.pntd.0007916

**Published:** 2019-12-02

**Authors:** Yujuan Yue, Dongsheng Ren, Xiaobo Liu, Yujiao Wang, Qiyong Liu, Guichang Li

**Affiliations:** 1 State Key Laboratory of Infectious Disease Prevention and Control, National Institute for Communicable Disease Control and Prevention, Chinese Center for Disease Control and Prevention, Beijing, People’s Republic of China; 2 School of Public Health, Jiamusi University, Jiamusi, People’s Repubulic of China; International Vaccine Institute, REPUBLIC OF KOREA

## Abstract

**Background:**

Scrub typhus, a serious public health problem in the Asia-Pacific area, is endemic in the “tsutsugamushi triangle” area. Scrub typhus has been widespread and has become a significant health concern in China. However, spatiotemporal patterns need to be investigated further.

**Objective:**

This study aims to explore spatiotemporal patterns, diffusion characteristics and regional distribution differences of scrub typhus cases in mainland China from January 2006 to December 2017.

**Method:**

Monthly cases of scrub typhus reported at the county level during 2006–2017 were obtained. Time-series analyses, spatial distribution analyses, spatial diffusion analyses, spatial autocorrelation analyses and space-time scan statistic analyses were used to explore spatiotemporal characteristics of scrub typhus.

**Results:**

A total of 121 251 scrub typhus cases were reported in 30 provinces (or municipalities) of mainland China during 2006–2017, which rose exponentially. There were seasonal characteristics from June to November for scrub typhus. Scrub typhus had been diffused from south, southwest, southeast and eastern coasts to center, north, northeast and northwest in mainland China. Scrub typhus occurrences were from point to surrounding regions, and from south to north every year. The peak periods of scrub typhus became longer and longer from north to southwest to south in mainland China. There existed a single peak in Southwest region and North region, respectively, but existed a bimodal peak for South region. Scrub typhus cases were clustered in Yunnan, Guangdong, Guangxi, Fujian and Anhui among June to November. The scrub typhus epidemics in Guangdong and Yunnan were the most serious.

**Conclusions:**

The results in this study can be guide targeted public health interventions against scrub typhus at the county level.

## Author summary

Scrub typhus is a bacterial zoonosis caused by *Orientia tsutsugamushi* and is popular in the Asia-Pacific area. Nowadays scrub typhus has been widespread and causes a considerable burden on public health and economy in China. We investigated spatiotemporal patterns, diffusion characteristics and regional distribution differences of scrub typhus cases in mainland China from 2006 to 2017. The majority of scrub typhus cases were reported in June to November, and 98.3% of the total scrub typhus cases during 2006–2017 were reported in South region, Southwest region and North region. Scrub typhus had been diffused in mainland China except Qinghai. Scrub typhus occurrences were from point to surrounding regions, and from south to north every year. The peak periods of scrub typhus turn longer, and there existed a single peak to a bimodal peak from north to south. Scrub typhus cases were clustered in Yunnan, Guangdong, Guangxi, Fujian and Anhui during June to November. The scrub typhus epidemics in Guangdong and Yunnan were the most serious.

## Introduction

Scrub typhus, a bacterial zoonosis caused by *Orientia tsutsugamushi* (*O*. *tsutsugamushi*), is characterized by fever, typical eschar or ulcer at the bite site, rash, headache, myalgia, cough, generalized lymphadenopathy, hepatosplenomegaly, nausea, vomiting, and abdominal pain [1–3]. Scrub typhus, which is transmitted occasionally to humans by the bites of infected chiggers (larval trombiculid mites) [4–5], can progress to multiple organ failure and even death in some cases [6–7]. Scrub typhus, a serious public health problem in the Asia-Pacific area [8], is endemic in the “tsutsugamushi triangle” area which includes Pakistan and Afghanistan in the west, far-eastern Russia and Japan in the north, and northern Australia in the south [9–13]. Scrub typhus threatens one billion people globally, and causes illness in one million people every year [9, 14–15]. There has been a drastic increase in both the frequency and geographic distribution of scrub typhus cases, which could signal the re-emergence of this neglected tropical disease [16].

Scrub typhus has been known in southern China for thousands of years [17]. Until the 1980s, scrub typhus cases primarily occurred in the regions south of Yangtze River including Zhejiang and Yunnan [18–19]. The first reported scrub typhus case in recent Chinese history was identified in Guangzhou City, Guangdong Province, 1948 [20]. While the first outbreak occurred in Shandong Province, 1986 [18, 21]. Previous studies have revealed that the incidence of scrub typhus has increased in a nationwide region of China [21–22]. In recent decades, scrub typhus has been widespread and has become a significant health concern in China [22,23].

Spatiotemporal analysis techniques have been widely applied in infectious disease control, prevention and scientific investigations [24–28]. Previous studies have identified clusters and geographic distribution variation of scrub typhus [29–30]. However, the nationwide characteristics of scrub typhus epidemics are unclear enough, and spatiotemporal patterns, diffusion characteristics and regional distribution differences should be investigated further. A detailed understanding of scrub typhus epidemics in China is needed to develop more effective public health responses to scrub typhus occurrences.

## Materials and methods

### Ethics statement

No human or animal samples were included in the research presented in this article, therefore ethical approval was not necessary for this research.

### Data collection and management

Scrub typhus is a vector-borne notifiable disease in China. The attending physicians are required by law to report to Chinese Center for Disease Control and Prevention (China CDC) through Chinese National Notifiable Infectious Disease Reporting Information System (CNNDS) (http://www.chinacdc.cn/). Scrub typhus report includes sex, age, occupation, national code of current address, and date of illness onset etc. Daily scrub typhus reports from Jan.1st 2006 to Dec. 31th 2017 were obtained from CNNDS. All scrub typhus cases were confirmed according to the diagnostic criteria issued by Chinese Center for Disease Control and Prevention, which include clinical diagnosis or laboratory diagnosis [27, 29–31]. Clinical manifestations are shown as high fever, lymphadenopathy, skin rash and eschar or ulcers. Laboratory diagnosis is as follows: a 4-fold or greater rise in serum IgG antibody titers between acute and convalescent sera by using indirect immunofluorescence antibody assay (IFA), or isolation of *O*. *tsutsugamushi* from clinical specimens, or detection of *O*. *tsutsugamushi* by polymerase chain reaction (PCR) in clinical specimens. Demographic data at the county level in 2010 was obtained from the sixth population census of the National Bureau of Statistics of China (http://www.stats.gov.cn/). The vector data of Chinese administrative divisions in 2017, which were used for geographical mapping, were provided by CNNDS (http://www.chinacdc.cn/). Data of the yearly rainfall zone and the climate zone, which were conducted according to Chinese meteorological station data during 1971–2000, were obtained from Research Center for Eco-Environmental Sciences, Chinese Academy of Sciences (http://www.rcees.ac.cn/).

There are four levels of administrative divisions in mainland China, namely province, city, county and town. In order to perform spatiotemporal analyses, scrub typhus cases were aggregated at the county level according to national codes of current addresses, and then were geocoded and matched to the county-level administrative boundaries using ArcGIS version 10.3 [32]. There are 31 provinces (or municipalities) comprised of 2 922 counties in mainland China, with population sizes ranging from 7,123 to 5,044,430 and geographic areas ranging in size from 5.4 to 197,346 square kilometers [27].

### Spatial, temporal and spatiotemporal analyses of scrub typhus

Time-series analyses for scrub typhus cases were conducted using IBM SPSS Statistics version 24.0 (IBM Corp., NY, USA). Spatial distribution analyses and Spatial emerging analyses of scrub typhus every year were conducted using Spatial Mapping in ArcGIS version 10.3.

Spatial diffusion analyses of scrub typhus during 2006–2017 were conducted using Global Polynomial Interpolation and Contour in ArcGIS version 10.3. When the power was set as 3 in this study, the trend surface of scrub typhus has the smallest root mean square. Contours, which indicated the invasive years of scrub typhus, were drawn based on the trend surface. The slope of the trend surface represented the reciprocal spread speed of scrub typhus from 2006 to 2017.

Global Moran’s I for global indication of spatial autocorrelation (GISA) reflects the similarity of attributes in spatial adjacent regions [33]. It is calculated on the basis of Z-test (P ≤ 0.05). It ranges from -1 to 1. The closer it approaches 1, the more aggregated the whole attributes are (High-value aggregation or Low-value aggregation). The closer it approaches -1, the more dispersed the whole attributes are. It is 0, which represents global random distribution. GISA was adopted to explore the global clustering characteristic of scrub typhus. Local Moran’s I for local indication of spatial autocorrelation (LISA) is a measure of the similarity or difference between the attribute of the observation unit and those of surrounding units.

The LISA accumulative map is drawn on the basis of Z-test (P ≤ 0.05) [33]. LISA was adopted to explore significant hotspots (High-High), coldspots (Low-Low), and outliers (High-Low and Low-High) of scrub typhus by calculating local Moran’s I index between the value in a given county and those in the surrounding counties. The significance level of clusters was determined by a Z score generated by comparison of the Local Moran’s I statistic for the average incidence in each county. A high positive Z score indicated that the surroundings had spatial clusters (High-High: high-value spatial clusters or Low-Low: low-value spatial clusters) and a low negative Z score indicated the presence of spatial outliers (High-Low: high values surrounded with low values or Low-High: Low values surrounded with high values)[34]. GISA analyses and LISA analyses can be realized by GeoDa version 0.9.

Kulldorff’s space-time scan statistic was used to explore the locations of high-risk space-time clusters, which can be realized by SaTScan version 9.3. The space-time scan statistic was defined by a cylindrical window with a circular (or elliptic) geographic base and with height corresponding to time [35]. Purely Spatial analysis scanning for clusters with high rates using the Discrete Poisson model was employed for average monthly scrub typhus cases in the counties. Circular scan windows were selected. The maximum spatial cluster size was set as 500 km. Likelihood ratio (LLR) tests were evaluated to determine the significance of identified clusters and P-values were obtained through Monte Carlo simulation after 999 replications. The null hypothesis of a spatiotemporally random distribution was rejected when the P-value was ≤0.05 [36]. Retrospective space-time analysis scanning for clusters with high rates using the Space-Time Permutation model was employed for monthly scrub typhus cases from 2006 to 2017 in the counties. Circular scan windows were selected. The maximum spatial cluster size was set as 500 km. Monte Carlo simulations after 999 replications were used to evaluate the significance of spatiotemporal clusters (p ≤0.05) [36].

## Results

### Time-series analyses for scrub typhus

**Fig 1:**
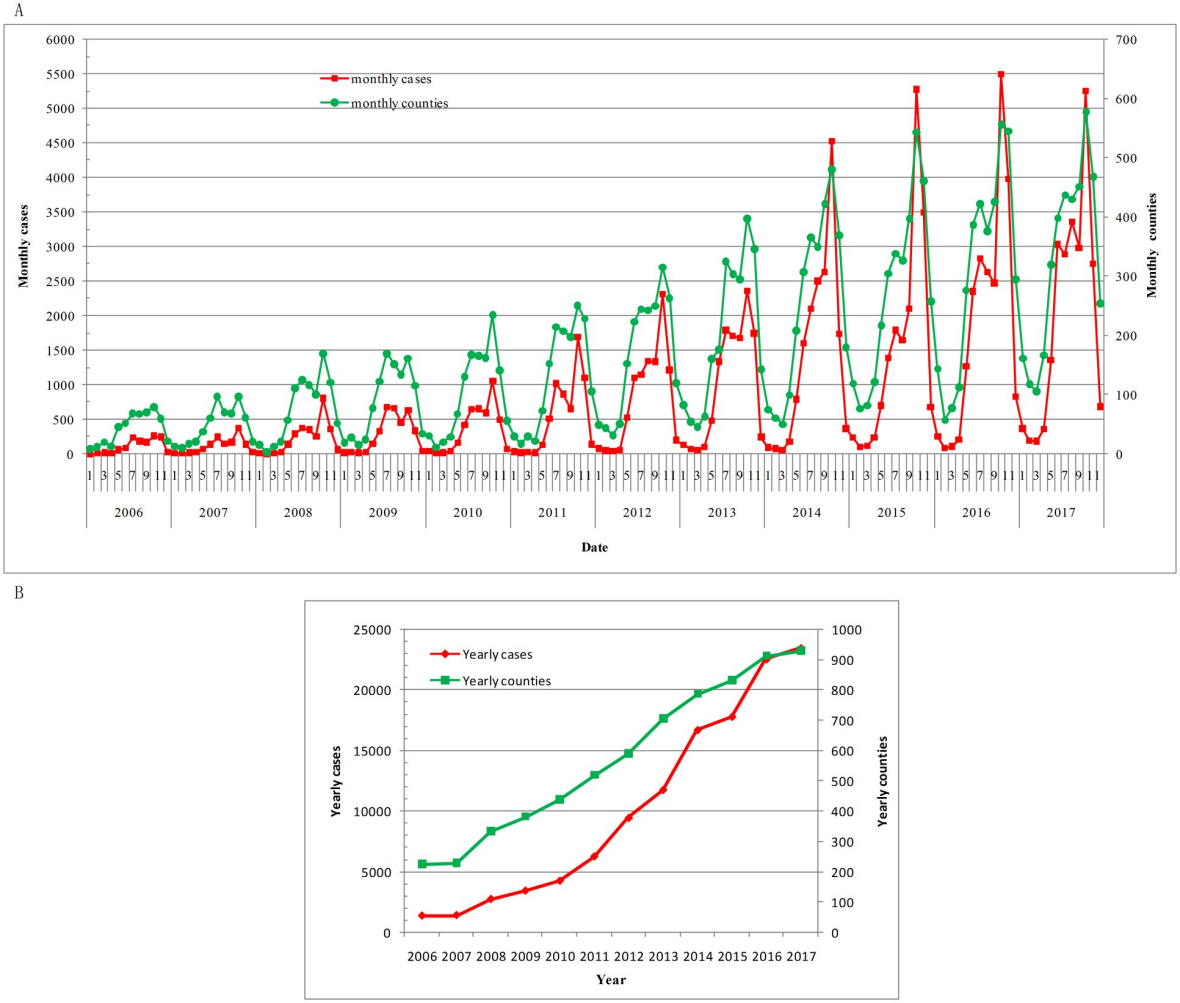
Time-series analyses of scrub typhus in mainland China, 2006–2017. A. Time-series mapping of monthly scrub typhus. B. Time-series mapping of yearly scrub typhus.

Scrub typhus showed seasonal pattern. The peak period of scrub typhus was from June to November each year, and the peaks of monthly cases and monthly counties were both in October every year (Fig 1(A)). Monthly cases and monthly counties in the same periods of each year increased steadily. The maximum monthly cases was 5 494 in October 2016. The maximum monthly counties was 579 counties in October 2017.

There were 121 251 cases in 2006–2017. The yearly cases and the yearly counties increased year by year (Fig 1(B)). The yearly cases increased from 1 375 to 23 474 in 2006–2017. The yearly counties increased from 226 to 930 in 2006–2017.

### Spatial distribution analyses for scrub typhus

**Fig 2:**
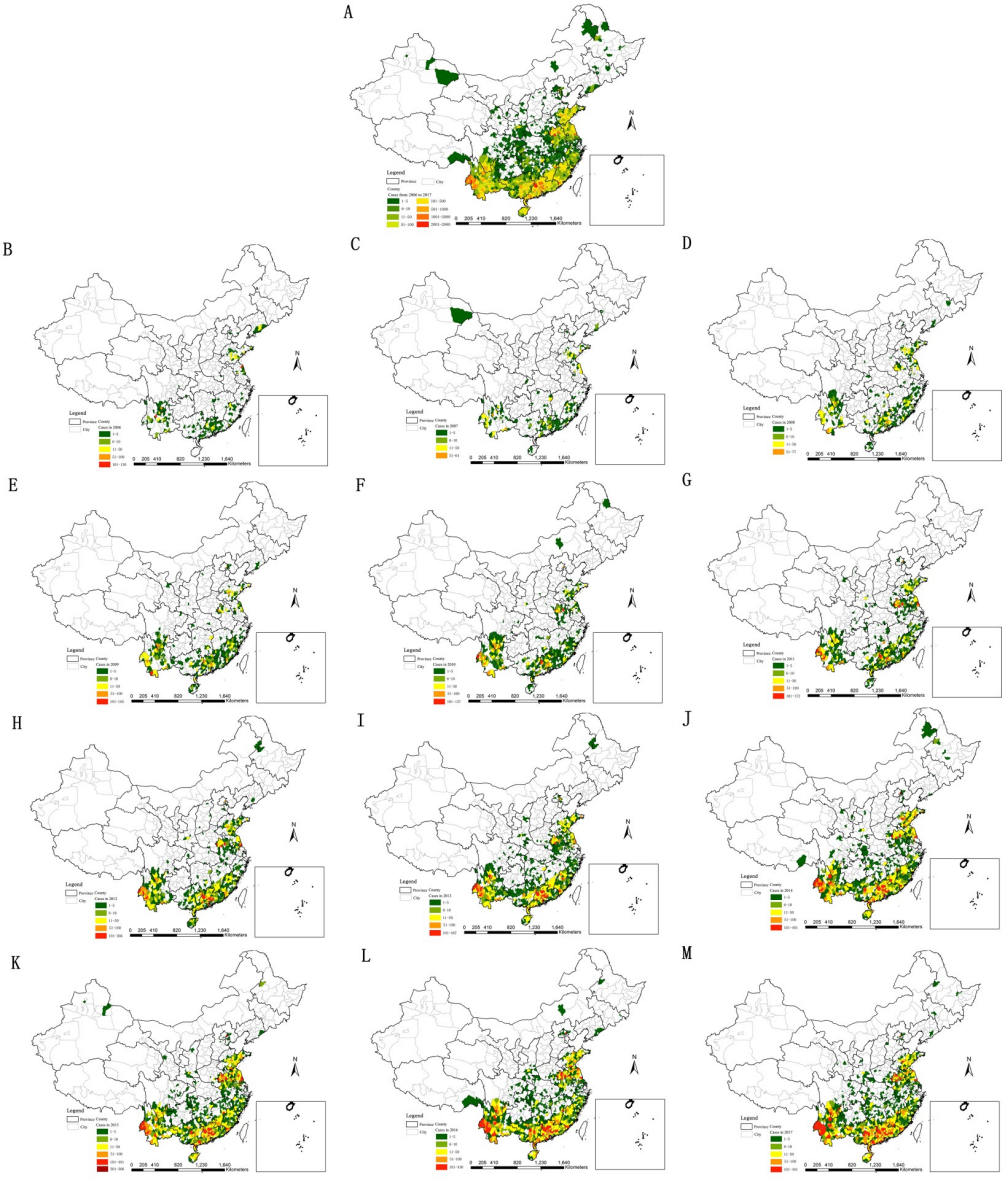
Scrub typhus distribution in 2006–2017. A. in twelve years; B. in 2006; C. in 2007; D. in 2008; E. in 2009; F. in 2010; G. in 2011; H. in 2012; I. in 2013; J. in 2014; K. in 2015; L. in 2016; M. in 2017. (The vector data of Chinese administrative divisions were provided by CNNDS. Figure 2 was created for this manuscript using ArcGIS.).

Scrub typhus occurred in 1 377 counties, 30 provinces (or municipalities) From 2006 to 2017. There were many scrub typhus cases among 100–2 000 in the county level in Yunnan, Sichuan, Hainan, Guangxi, Guangdong, Jiangxi, Fujian, Jiangsu, Anhui and Shandong (Fig 2(A)), where the epidemic situations were serious. Scrub typhus occurred in the total area of Hainan, Guangdong and Fujian. There were more than 2 000 cases in four counties, respectively, namely Longling County in Yunnan Province, Huaiji County and Guangning County in Guangdong Province, and Yingshang County in Anhui Province. The counties with more than 100 scrub typhus cases every year were located in Yunnan, Sichuan, Hainan, Guangxi, Guangdong, Jiangxi, Fujian, Jiangsu, Anhui and Shandong too (Fig 2(B)–2(M)).

### Spatial diffusion analyses for scrub typhus

**Fig 3:**
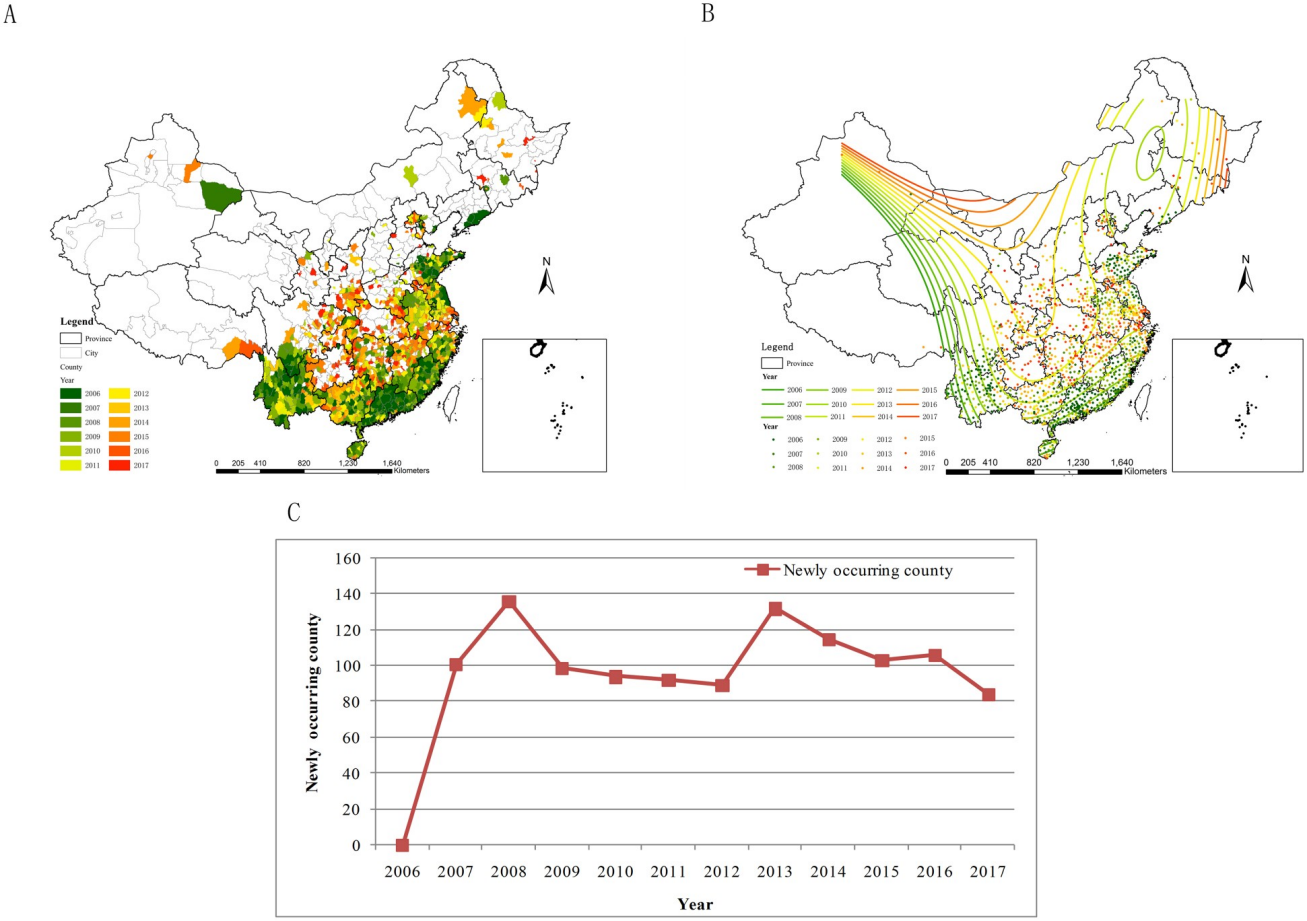
Spatial diffusion analyses of scrub typhus in 2006–2017. A. Spatial diffusion mapping of scrub typhus in 2006–2017. B. Spatial diffusion of scrub typhus by Trend. C. Newly emerging Counties of scrub typhus along the years. (The vector data of Chinese administrative divisions were provided by CNNDS. Figure 3 was created for this manuscript using ArcGIS.).

According to Spatial mapping and Trend analyses, scrub typhus occurred in mainland China except Qinghai. Scrub typhus had been diffused from south, southwest, southeast and eastern coasts to center, north, northeast and northwest in mainland China (Fig 3(A) and 3(B)). In 2006, there were 226 counties related with scrub typhus. There were more than 80 new counties every year where scrub typhus happened compared with all the scrub typhus counties in previous years (Fig 3(C)). Among them, there were more than 130 new counties with scrub typhus in 2008 and 2013, respectively.

**Fig 4:**
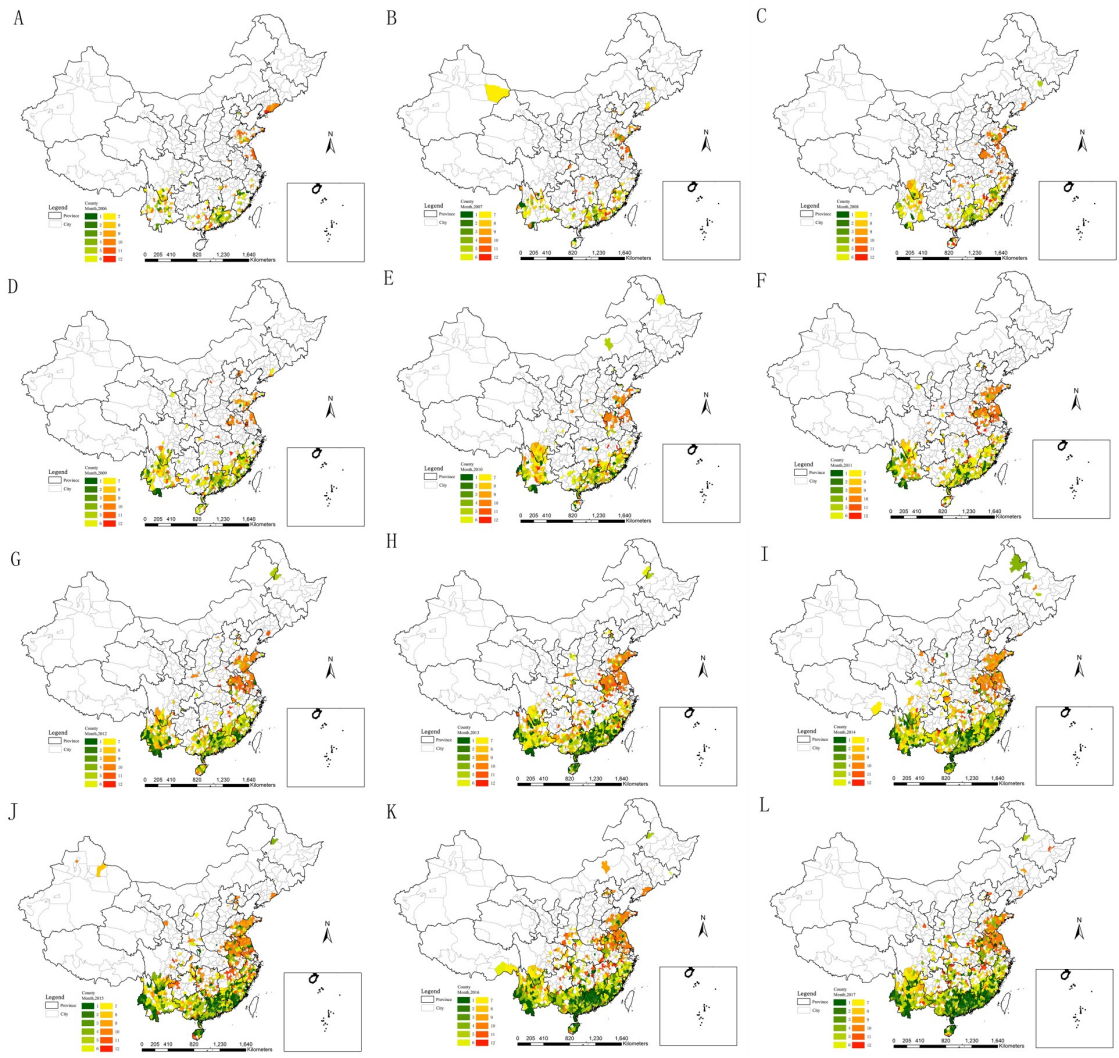
Spatial emerging analyses for monthly scrub typhus during 2006–2017. A. in 2006; B. in 2007; C. in 2008; D. in 2009; E. in 2010; F. in 2011; G. in 2012; H. in 2013; I. in 2014; J. in 2015; K. in 2016; L. in 2017. (The vector data of Chinese administrative divisions were provided by CNNDS. Figure 4 was created for this manuscript using ArcGIS.).

Scrub typhus occurrences were from point to surrounding regions, and from south to north every year (Fig 4). Scrub typhus cases occurred mainly in Sichuan, Yunnan, Guangxi, Guangdong, Fujian, Zhejiang, Jiangxi, Hunan and Hainan from January to July, while emerged relatively dispersedly from August to September and November to December. Scrub typhus cases were concentrated mainly in Jiangsu, Anhui and Shandong in October.

### Spatial autocorrelation analyses for scrub typhus

**Fig 5:**
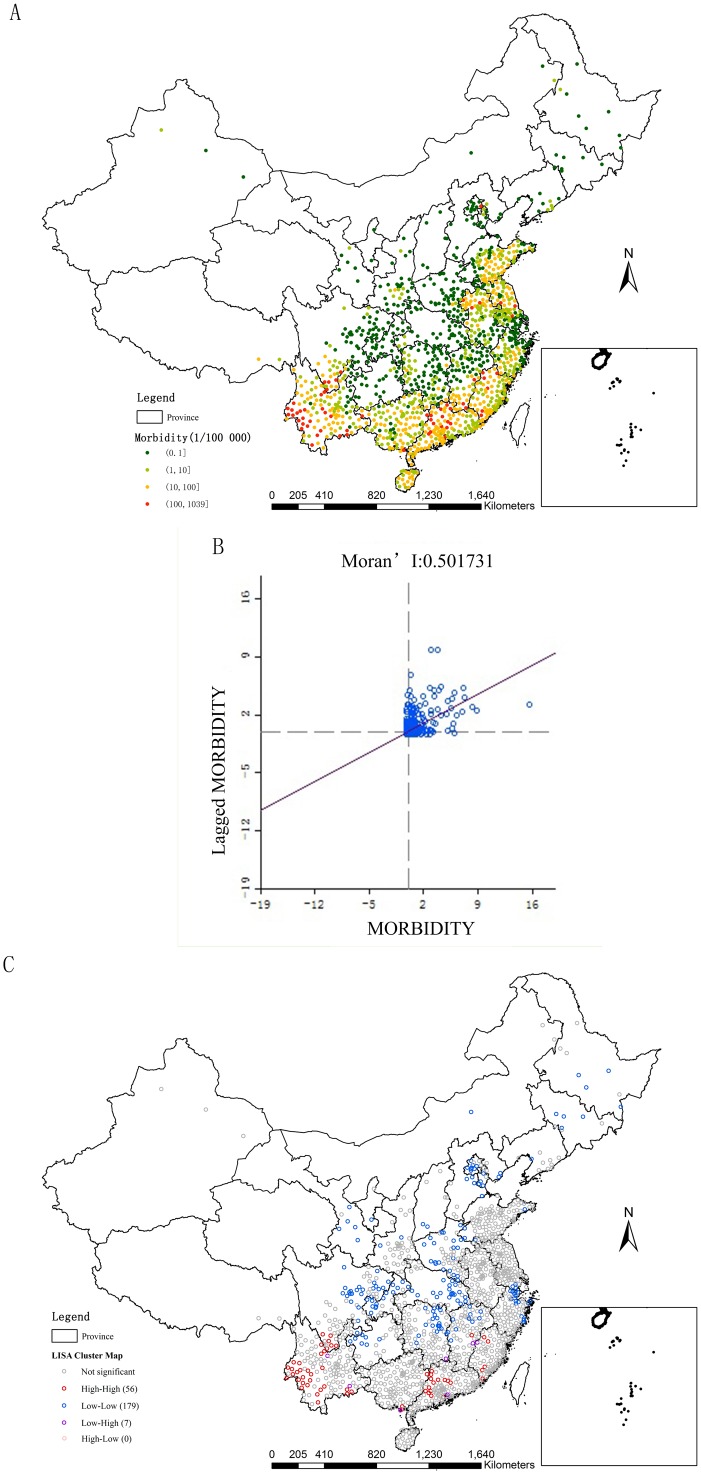
Spatial autocorrelation analyses. A. Scrub typhus morbidity in twelve years. B. GISA. C. LISA. (The vector data of Chinese administrative divisions were provided by CNNDS. Figure 5 was created for this manuscript using ArcGIS.).

The counties with scrub typhus morbidity More than 100 (1/10^5^) were located in some counties of Yunnan, Sichuan, Guangxi, Guangdong, Jiangxi, Fujian, Anhui, Jiangsu, and Beijing (Fig 5(A)). The value of Global Moran’s I on the basis of statistical significance was 0.501, which indicated that scrub typhus morbidity had strong spatial clustering characteristics of high or low values (Fig 5(B)).

The LISA accumulative map of scrub typhus morbidity was drawn on the basis of Z-test (P ≤ 0.05). 56 counties were clustered in the High-High regions. Most of them were in Yunnan and Guangdong, and the others were in Sichuan, Guangxi, Fujian and Jiangxi. 179 counties were located in the Low-Low regions, mainly in the central provinces as Hunan, Hubei, Jiangxi, Sichuan, Chongqing and Henan, the Yangtze River Delta region and the Beijing-Tianjin-Hebei region (Fig 5(C)).

### Space-time scan statistic analyses for scrub typhus

**Fig 6:**
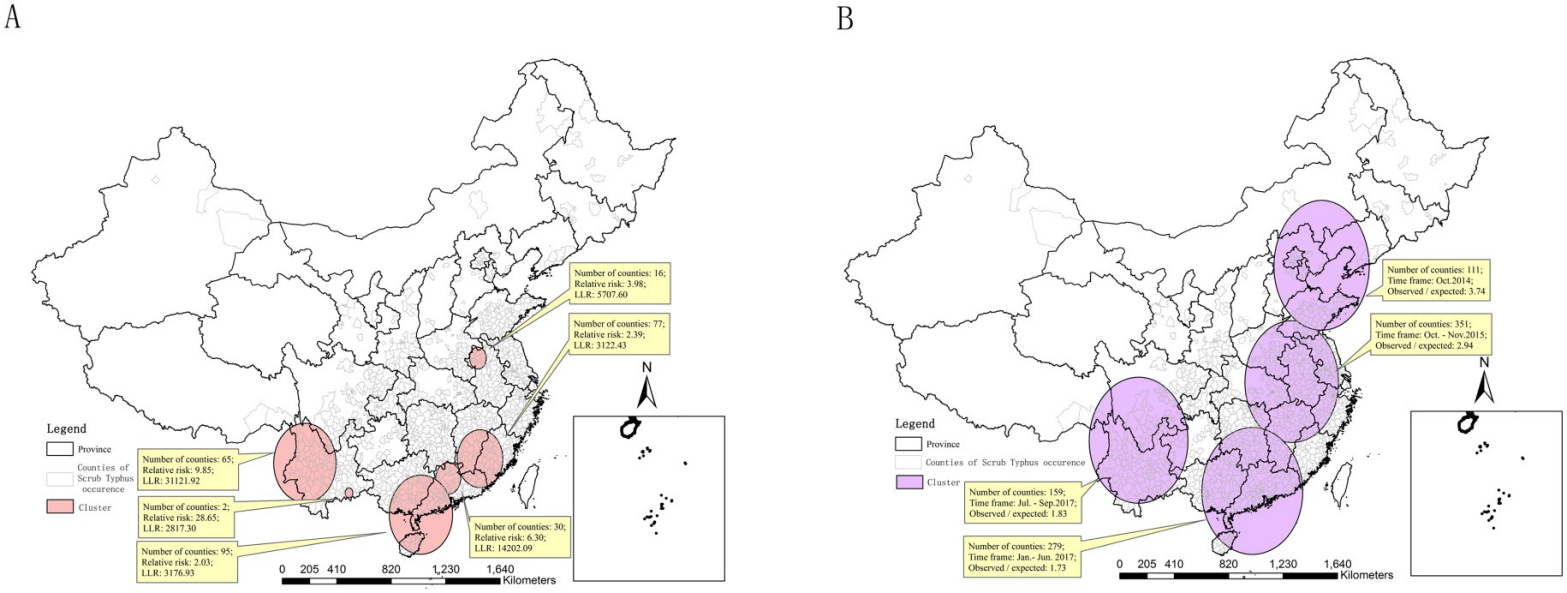
Space-time scan statistic analyses for scrub typhus. A. the purely spatial analysis scanning in 12 years. B. the retrospective space-time analysis scanning from 2006 to 2017. (The vector data of Chinese administrative divisions were provided by CNNDS. Figure 6 was created for this manuscript using ArcGIS.).

According to the results of purely spatial analysis scanning for scrub typhus, there are 5 spatial clusters with LLR greater than 1 000, which were in southwestern China as Yunnan, southern China as Guangxi, Guangdong and Fujian, and Northern China as Anhui (Fig 6(A)).

According to the results of retrospective space-time analysis scanning for scrub typhus, there were 4 spatiotemporal clusters. One cluster located in 159 counties in Yunnan, Guizhou and Sichuan from July to September 2017. One cluster located in 279 counties in Guangdong, Guangxi, Hunan, Jiangxi and Fujian from Jan. to Jun. 2017. One cluster located in 351 counties in Jiangsu, Anhui, Zhejiang, Jiangxi, Hubei, Henan and Shandong from Oct. to Nov. 2015. One cluster located in 111 counties in Shandong and the Beijing-Tianjin-Hebei region in Oct. 2014(Fig 6(B)).

### Scrub typhus analyses in key regions

**Fig 7:**
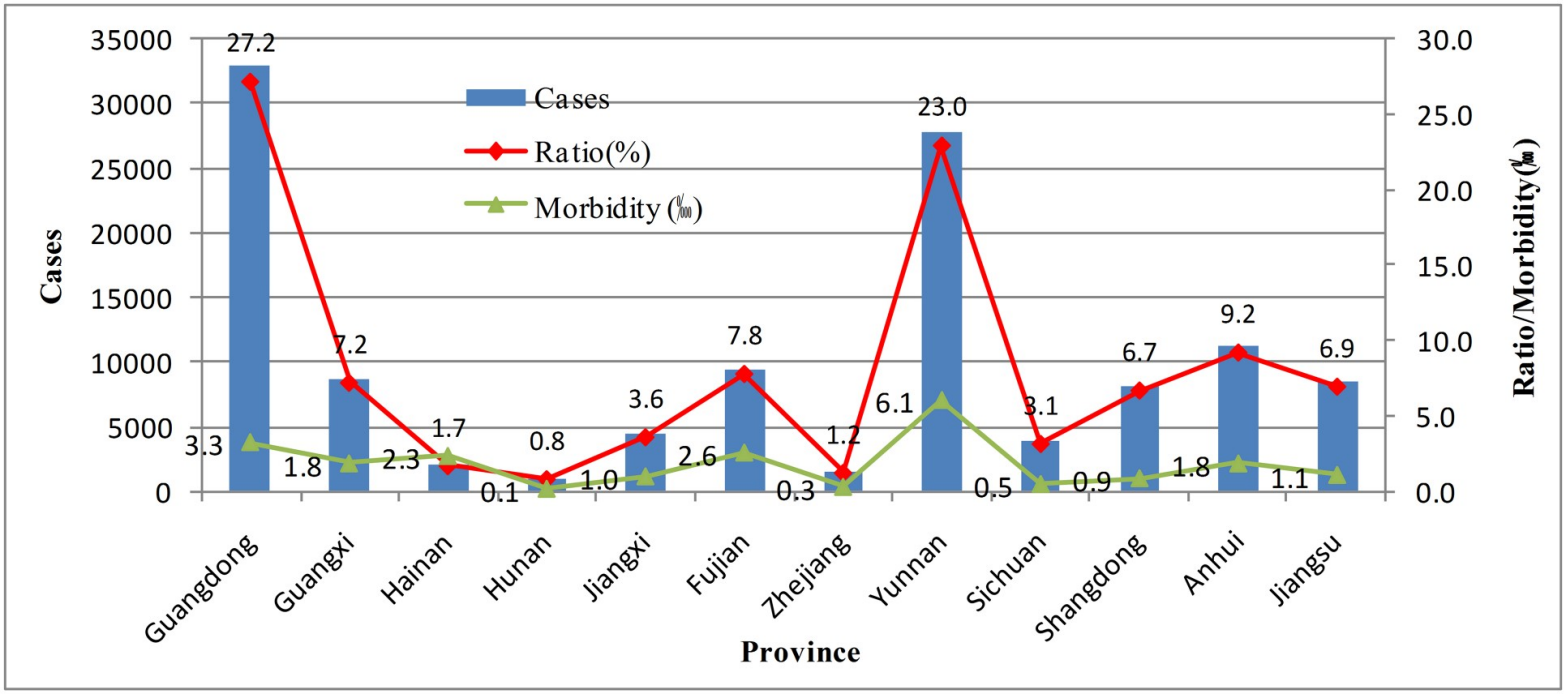
Scrub typhus cases, the case ratio and the morbidity in key regions in 12 years.

Considering spatiotemporal characteristics of scrub typhus and geographical locations such as the similar latitudes, 12 provinces of Hainan, Guangdong, Guangxi, Yunnan, Sichuan, Hunan, Jiangxi, Fujian, Zhejiang, Jiangsu, Anhui and Shandong, where 98.3% of the total scrub typhus cases during 2006–2017 occurred, were considered as key regions of scrub typhus. The total scrub typhus cases were over 100 at the county level in the most areas of the key regions during 2006-2017(Fig 2(A)). The total morbidity in Yunnan, Guangdong, Fujian and Hainan ranked the top four (Fig 7). The scrub typhus epidemics in Guangdong and Yunnan were the most serious.

**Fig 8:**
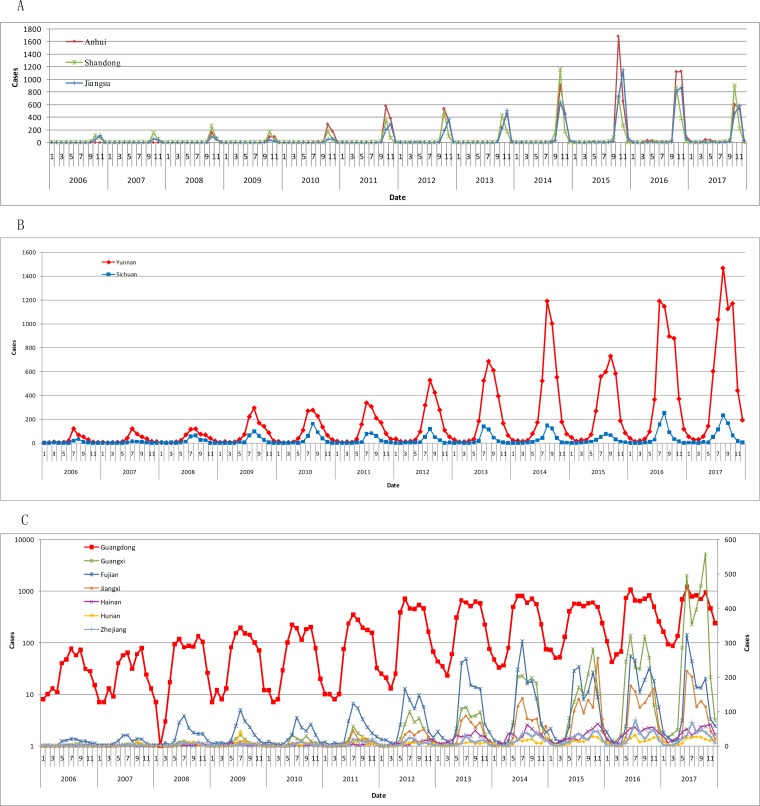
Time-series analyses of scrub typhus in key regions in 2006–2017. A. North region. B. Southwest region B. C. South region.

According to the time-series mapping repeatedly, the key regions were divided into three sub regions, and each one had its own features (Fig 8). North region concluded Shandong, Anhui and Jiangsu. Southwest region concluded Yunnan and Sichuan. South region concluded Hainan, Guangxi, Guangdong, Fujian, Hunan, Jiangxi and Zhejiang (Table 1).

**Table 1:** The onset characteristics of scrub typhus in the key sub regions.

Region\Characteristics	Onset characteristics	Summary	Climate and rainfall zones
North region: Shandong, Anhui, Jiangsu	The peak period of scrub typhus onset, with a single peak, was from October to November every year. The monthly cases in each province in peak periods could reach more than 1000 in recent years. The maximum cases were 1684, which originated from Anhui Province, October 2015. There were a few scrub typhus cases outside peak periods, and the monthly cases in each province were generally less than 10.	Short peak period from October to November, high outbreak, single peak.	Located in the warm temperature zone. With the yearly rainfall among 800-1600mm.
Southwest region: Yunnan, Sichuan	The peak period of scrub typhus onset, with a single peak, was from July to October every year. In recent years the monthly maximum cases in Yunnan in peak periods could reach nearly 1500.	Middle peak period from July to October, high outbreak, single peak.	Located in the subtorrid zone. With the yearly rainfall among 800-1600mm.
South region: Hainan, Guangxi, Guangdong, Fujian, Hunan, Jiangxi, Zhejiang	The peak period of scrub typhus onset, with double peaks, was from May to December every year. In recent years Guangdong had the most cases, with a peak monthly cases of about 1000. Guangxi had a peak monthly cases of nearly 600. Fujian had a peak monthly cases of nearly 300. And Jiangxi had a peak monthly cases of nearly 200. In addition, Hainan, Hunan and Zhejiang have fewer cases, and the monthly cases were controlled within 100 cases, respectively.	Long peak period from May to November, sustained outbreak, bimodal peak.	Located in the subtorrid zone or the torrid zone. With the yearly rainfall among 800-1600mm or more than 1600mm.

**Fig 9:**
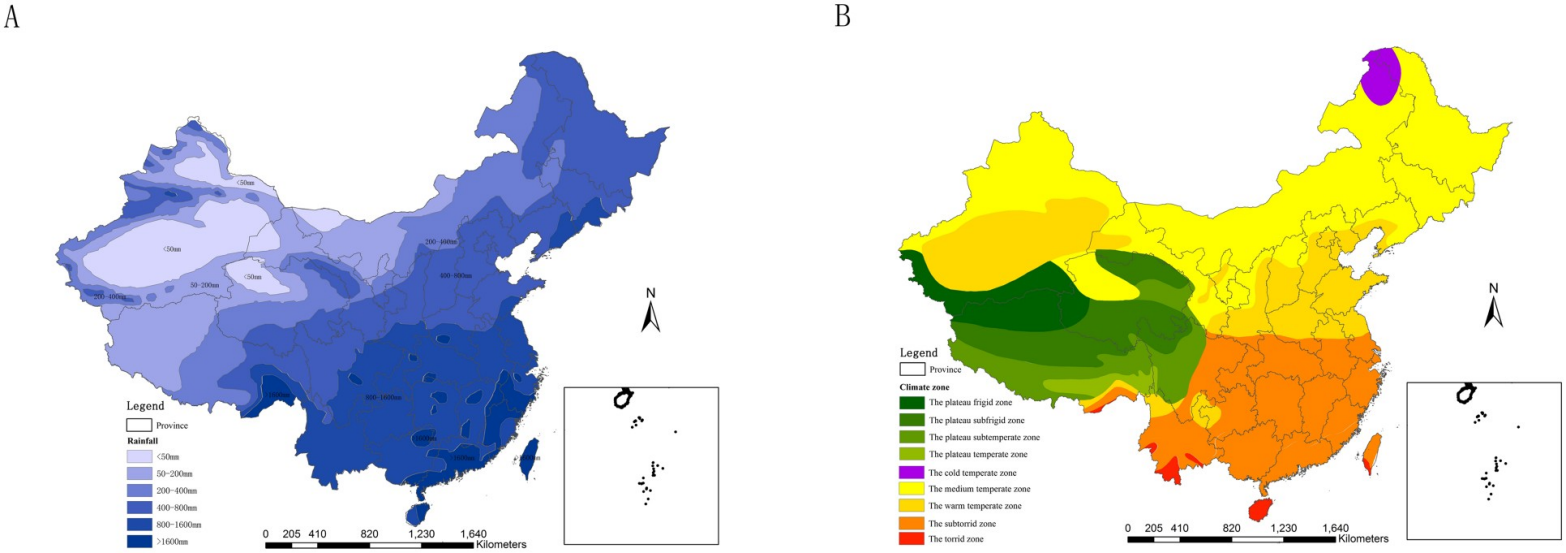
The yearly rainfall zone and the climate zone. A. the yearly rainfall zone. B. the climate zone. (Data of the yearly rainfall zone and the climate zone were obtained from Research Center for Eco-Environmental Sciences, Chinese Academy of Sciences. Figure 9 was created for this manuscript using ArcGIS.).

Based on the time-series analyses of scrub typhus (Fig 8) and yearly rainfall zone and climate zone (Fig 9), the onset characteristics of scrub typhus in key sub regions were summarized (Table 1). North region had short peak period, high outbreak and single peak. Southwest region had middle peak period, high outbreak and single peak. South region had long peak period, sustained outbreak, bimodal peak. The yearly rainfall in most of the key regions ranged from 800 mm to 1 600 mm. The yearly rainfall in most of the areas in Hainan, Guangdong, Fujian and Zhejiang were more than 1 600 mm. Most of the key regions belong to the torrid and subtorrid zone. Yunnan locates in torrid and subtorrid zone, with the yearly rainfall of 800–1 600 mm. Guangdong locates in the subtorrid zone, with the yearly rainfall more than 1 600 mm in its most regions.

## Discussion

There were 121 251 scrub typhus cases in 2006–2017, which located in 1 377 counties, 30 provinces (or municipalities) except Qinghai Province. There were seasonal characteristics from June to November for scrub typhus, which was similar to the results in the previous studies that the majority of cases (86.8%) occurred during May-October, Guangzhou [37]. Scrub typhus cases and counties increased exponentially year by year. There were 23 474 scrub typhus cases and 930 counties in 2017. Scrub typhus had been diffused from south, southwest, southeast and eastern coasts to center, north, northeast and northwest in mainland China. Scrub typhus occurrences were from point to surrounding regions, and from south to north every year. With the rapid development of society and economy, climate change, population movement, better recognition by health care professionals and ever-improving detection techniques, both sporadic cases and disease outbreaks began to be identified in the northern provinces of Shandong, Jiangsu, Tianjin and Beijing, as well as the emergence of new natural foci in the past three decades [18, 38–40]. Scrub typhus is widespread in mainland China, where the incidence has increased rapidly in recent years [23]. Without appropriate treatment, the case fatality rate of scrub typhus can reach 30% or even higher [8]. It is also a travel associated disease [14] and of great importance among military personnel [41–42]. To date, there is still no effective and reliable human vaccine against scrub typhus and no point-of-care diagnostics available [12, 43]. Therefore, Attention should be paid to scrub typhus in mainland China, and effective prevention and control should be taken.

The key regions, divided as Southwest region, South region and North region (Table 1), accounted for 98.3% of the total scrub typhus cases during 2006–2017 in this research, with scrub typhus cases over 100 in most of the counties (Fig 2(A)). Different geographical, climatic and environmental factors lead to different epidemic characteristics of scrub typhus in the sub regions. The peak periods of scrub typhus became longer and longer from north to southwest to south in mainland China. There existed a single peak in Southwest region and North region, respectively, but existed a bimodal peak for South region. Guangdong was with a bimodal peak in human cases typically occurring from May to November [44]. The annual rainfall rose from 800 mm to more, and the climate zone turned from the warm temperature zone to the sub-torrid zone to the torrid zone from north to southwest to south (Figs 8 and 9). Scrub typhus are correlated closely with climate [45] such as temperature and rainfall [46] and environmental factors. Transmission of scrub typhus varies across seasons and geographical areas in China [23]. Scrub typhus incidence was positively correlated with the percentage of shrub, and temporal variation in temperature and precipitation in China [27]. Atmospheric pressure and relative humidity with lags of 1 or 2 months, distributions of broadleaved forest and rural township were identified as determinants for the spatiotemporal distribution of scrub typhus [37]. Relative humidity, rainfall, DTR, MEI and rodent density were associated with the incidence of scrub typhus [23]. Temperature, duration of sunshine, and rainfall were positively associated with scrub typhus incidence, while atmospheric pressure was inversely associated with scrub typhus incidence [31].

Scrub typhus epidemics in South region and Southwest region were more popular (Fig 8). Several high-risk regions for scrub typhus were identified in southwest, southern, and middle-east China [27]. Most of the High-High regions located in Yunnan and Guangdong, and Low-Low regions mainly located in the central China, the Yangtze River Delta region and the Beijing-Tianjin-Hebei region (Fig 5(C)). Scrub typhus cases were clustered in Yunnan, Guangdong, Guangxi, Fujian and Anhui among June to November (Fig 6). The scrub typhus epidemics in Guangdong and Yunnan, which located in South region and Southwest region, were the most serious, with about 30 000 cases during 2006–2017, respectively. Scrub typhus is becoming the most common vector born disease in Guangzhou, southern China [31]. Scrub typhus in Guangzhou is of the summer-type and is more virulent than the autumn-type scrub typhus which is endemic in Northern China [30].

There are large cases and wide spatial distributions including all the provinces (or municipalities) except Qinghai Province for scrub typhus in mainland China in recent years, and scrub typhus has become one of the most important vector-borne infectious diseases. Therefore, local governments should focus on the investigations and studies of scrub typhus foci, study the causes of the rising trend of scrub typhus in recent years, and provide decision-making and support for the prevention and control of scrub typhus in China further. This study analyzed the temporal, spatial and spatiotemporal characteristics of scrub typhus during 2006–2017 comprehensively and detailedly. We will focus on the relationships between the potential influential factors as geographical, climatic and environmental factors and scrub typhus, explore the reasons for the rise and spread of scrub typhus in mainland China, and predict the incidence trend of scrub typhus further in the next step.
